# A Method for Detecting Dynamic Mutation of Complex Systems Using Improved Information Entropy

**DOI:** 10.3390/e21020115

**Published:** 2019-01-27

**Authors:** Bin Ju, Haijiao Zhang, Yongbin Liu, Donghui Pan, Ping Zheng, Lanbing Xu, Guoli Li

**Affiliations:** 1 College of Electrical Engineering and Automation, Anhui University, Hefei 230601, China; 2 National Engineering Laboratory of Energy-Saving Motor & Control Technology, Anhui University, Hefei 230601, China; 3 College of Mathematical Sciences, Anhui University, Hefei 230601, China

**Keywords:** nonlinear dynamics, probability mass function, improved information entropy, rolling bearing, fault diagnosis

## Abstract

In this study, a nonlinear analysis method called improved information entropy (IIE) is proposed on the basis of constructing a special probability mass function for the normalized analysis of Shannon entropy for a time series. The definition is directly applied to several typical time series, and the characteristic of IIE is analyzed. This method can distinguish different kinds of signals and reflects the complexity of one-dimensional time series of high sensitivity to the changes in signal. Thus, the method is applied to the fault diagnosis of a rolling bearing. Experimental results show that the method can effectively extract the sensitive characteristics of the bearing running state and has fast operation time and minimal parameter requirements.

## 1. Introduction

Mechanical equipment such as a rolling bearing often causes irregular vibrations due to the influence of impacts, variable loads, and friction during operation [1,2]. Therefore, the acquired signals usually have non-stationary and nonlinear characteristics. The bearing is generally analyzed using a combination of various characteristic parameters to evaluate the health of the bearing for the purpose of accurately identifying its state. However, the regularity and sensitivity of the states reflected by various parameters are usually different [3,4,5]. Meanwhile, the correlation of multiple characteristic parameters often causes difficulties in the analysis. Moreover, using too many feature parameters will affect the computational cost and accuracy of the pattern classification [6]. In addition, achieving satisfactory results is difficult when a traditional linear signal processing method is applied to the feature extraction and recognition of nonlinear signals. The development of nonlinear theory has enabled researchers to propose a series of nonlinear signal processing methods, such as support vector machines [7,8], Kernel Principal Component Analysis [9], Lyapunov exponent [10], symbolic dynamics [11], and Hilbert–Huang transform methods [12,13]. These nonlinear signal processing methods have been used successfully to extract and identify the state characteristics of rolling bearings. These applications have positive importance for exploring new methods of monitoring and fault diagnosis of bearing conditions.

In recent years, an increasing number of nonlinear analysis methods have been applied to fault diagnosis, such as sample, fuzzy, and multi-scale entropies. The first time that entropy was published for health status monitoring was in 1998 by Basaran and Yan [14]. They utilized the entropy production as the sole measure for evaluating the damage mechanics of solder joints [15]. Sosnovskiy et al. proposed a generalized theory of evolution for mechanothermodynamic systems based on the concept of tribo-fatigue entropy [16]. Yan et al. [17] applied approximate entropy to the health status monitoring of mechanical systems and achieved good monitoring results. Yang et al. [18] combined fuzzy entropy with a variable predictive mode-based class discriminate and applied it to fault diagnosis of rotor systems. Reference [19] applied multi-scale entropy to fault diagnosis of rolling bearings. However, the similarity measure in sample entropy is prone to mutation, and fuzzy entropy calculation is time-consuming; moreover, multi-scale entropy is based on sample entropy [20,21,22]. These algorithms are greatly affected by the non-stationary nature of time series and are unsuitable for online monitoring. To solve this issue, Bandt et al. [23] proposed a method called permutation entropy (PE) for measuring the complexity of one-dimensional time series. This algorithm features simple calculation, strong anti-noise ability, and short time series and is suitable for online monitoring. The method can effectively detect dynamic catastrophes of complex systems and is widely applied in various fields [24,25,26,27]. However, the calculation of PE depends on the selection of embedded dimension *m* and delay time τ, which affect the effectiveness of PE detection [28].

The occurrence of mechanical faults is a process of mutation. The singularity of signal mutation is important information reflecting mechanical faults. Thus, detecting the signal mutation becomes a key point in studying the operating state of mechanical equipment [29]. The Fourier transform is an effective method to detect signal mutation and is widely applied in various fields [30,31,32]. However, the Fourier transform method can only determine the overall nature of sudden changes in signal and cannot determine the exact location of these changes in a time domain. With the development of dynamics theory, scholars have proposed a series of methods for detecting signal mutation, such as dynamic transformation regression, nonlinear coherence analysis, and related probability and statistical methods [33,34,35,36]. These algorithms can effectively solve the problem but have complex calculation processes and large calculation demands. 

To overcome the shortcomings of the existing technology, this study proposes a new method called improved information entropy (IIE) on the basis of constructing a special probability mass function for the normalized analysis of Shannon entropy to measure the complexity of one-dimensional time series. Unlike the PE, the IIE has lesser parameter selection and shorter calculation time. The simulation results show that the IIE can be used as a signal parameter to distinguish different kinds of signals and can effectively detect the mutation in a signal. Therefore, we introduce IIE into the fault signal diagnosis of a bearing. The experimental results show that the IIE can effectively judge the occurrence point and degree of bearing fault.

The rest of the paper is organized as follows. In Section 2, the theoretical background of IIE is introduced, and the characteristics of IIE applied on typical signals are analyzed. In Section 3, two experimental cases are proposed for verifying the effectiveness of the IIE on the fault diagnosis of rolling bearings. In Section 4, the conclusions are elaborated.

## 2. Methodology

### 2.1. Basic Definition of the Proposed Improved Information Entropy

A set of time series composed of *N* points is considered, and these variables can be written as $\left\{x_{i},i=1,2,3,\cdots ,N\right\}$. The Shannon entropy can be used to quantify the uncertainty of the given time series. In this calculation of the entropy, the extraction of the probability mass function (PMF) is an essential step. In fact, if there are some outliers or mutations in a given time series, they will deviate greatly from the mean of the sequence, and the probability of occurrence is very low. In order to highlight and evaluate the effect of the deviation degree on the entropy calculation, we construct the following PMF *p*(*i*) for each data point $x_{i}$:

(1) $$p(i)=e^{-\frac{{\left(x_{i}-\mu \right)}^{2}}{2\sigma^{2}}},\;i=1,2,\cdots ,N$$

where *μ* and *σ* are the mean and standard deviations of the time series, respectively, and can be calculated as follows:

(2) $$\mu =\frac{1}{N}\sum_{i=1}^{N}x_{i},$$

(3) $$\sigma =\sqrt{\frac{1}{N-1}\sum_{i=1}^{N}{\left(x_{i}-\mu \right)}^{2}}.$$

It is worth noting that the PMF *p*(*i*) constructed in Equation (1) has the following characteristics:

(1) 0≤p(i)≤1.

(2) p(i) will approximate to 1 when $x_{i}$ is close to μ, and then −ln*p*(*i*) will approach 0.

(3) *p*(*i*) will approximate to 0 when $x_{i}$ is away from μ, and then −ln*p*(*i*) will approach infinity.

It is necessary to construct such PMF *p*(*i*) for the following calculation of the entropy to reflect the mutation characteristics of a time series. If there occurs an outlier or a mutation $x_{i}$ in the time series, it is far away from the mean of the sequence. In this case, the *p*(*i*) will be close to 0, using the aforementioned constructed PMF. Therefore, the effect of the mutation will be amplified in the calculation of the entropy. The Shannon entropy *H* is a non-negative index to measure the complexity of time series, and can be calculated as follows:

(4) $$H=-\sum_{i=1}^{N}p(i)\ln(p(i)).$$

By normalizing the entropy *H* above, a parameter of improved information entropy (IIE) is defined to quickly detect the dynamic mutation of one-dimensional time series. The IIE is given by

(5) $$\mathrm{IIE}=-\ln(\frac{H}{N}),$$

where *N* is the length of the time series mentioned above.

**Figure 1:**
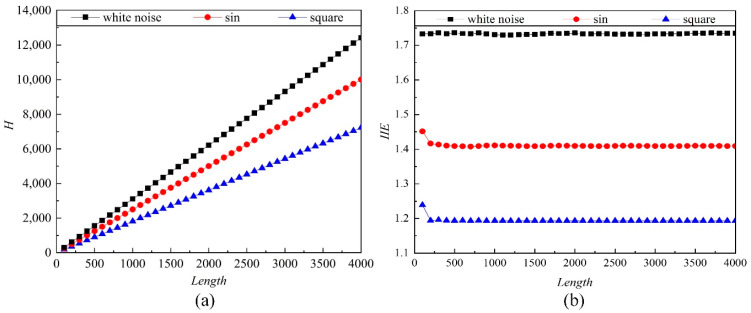
The effect of data length on the entropy values of *H* (**a**) and improved information entropy (IIE) (**b**) of different signals.

This normalization processing is done in order to make the calculated entropy weakly correlated with the length *N* since the parameter of *N* directly influences the calculation speed. So next, we verify the effect of the data length *N* on IIE below. As shown in Figure 1, the entropy values of *H* and IIE calculations are both performed on sine signals, square wave signals, and white noise of lengths of 100, 200, 300, ..., 4000. According to the calculation results, the values of *H* increase with the length N, which is not suitable for the entropy comparison for different signals with different lengths. Whereas, after the normalization process, the calculated entropy values of IIE weakly correlate with the length *N* as shown in Figure 1b. The results show that the three signals fluctuate at the beginning because the initial time series is too short and the randomness of the signals is insufficiently stable. However, as the time series length increases, the IIE values of the three signals are stable near a constant value. Therefore, the IIE has less dependence on the length of the time series. For the difference in the IIE values of different kinds of signals, a detailed analysis will be carried out in the next section. 

### 2.2. The IIE Characteristics of Typical Signals

**Figure 2:**
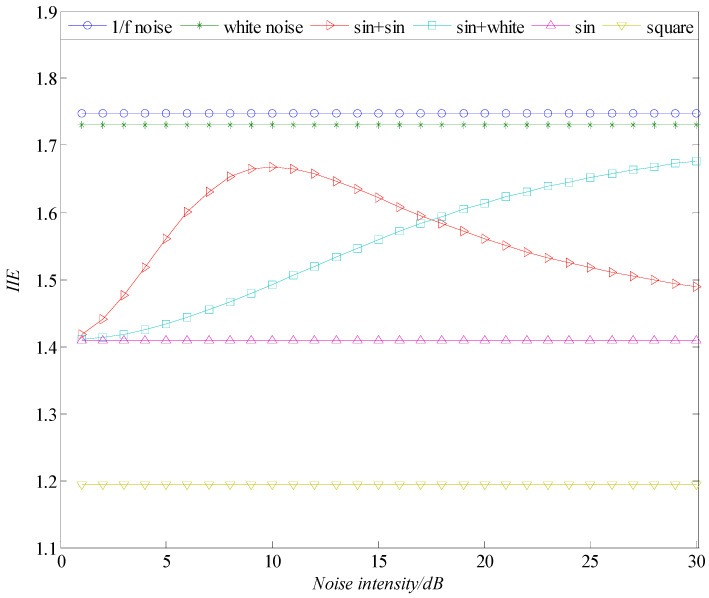
Variation in IIE of several typical signals with different noise intensities.

On the basis of the definition of the IIE above, we calculate the IIE of several typical signals, as shown in Figure 2. These typical signals include a sine wave, square wave, white noise, 1/f noise, and sine signal superimposed with different noise signals. Each calculated IIE value corresponds to a time series length of 2048. For a single signal (sine wave, square wave, white noise, and 1/f noise), we consider it as a noise signal and calculate the IIE value under different signal intensities. For sine signals superimposed with different noise signals, the intensity of the sine signal is set to 17 dB, and the IIE variation with superimposed noise under different intensities is calculated.

As shown in Figure 2, the entropy value for a single signal is very stable with the increase in the signal intensity. This result is due to the fact that the degree of randomness is definite for a certain single signal; the noise signal has the largest degree of randomness, followed by the sine wave, whereas the square signal has the smallest degree of randomness. The randomness can also be reflected by the IIE values of these single signals; the entropy value of the square wave is the smallest, the sine wave is in the middle, and the noise signal is the largest. Thus, the IIE can be a characteristic parameter that reflects the degree of randomness of a time series and has a certain degree of discrimination for different signals. A high value of entropy indicates high randomness of the signal and vice versa. The IIE values of broadband and narrowband noise signals are very high and have little difference compared with those of the white noise and 1/f noise. The randomness of the two noise signals is large but inconsiderably differ. Therefore, only white noise is selected when calculating the IIE value of the sine signal superimposed with different noises. In the actual test environment, some regular noise signals may be coupled. Thus, the IIE analysis of sine signal superimposed with sine noise is also conducted.

Figure 2 shows the IIE values of a sine signal superimposed with sine and white noises of different intensities. The amount of information contained in the signal increases due to the superposition of different signals. As a result, the value of entropy is higher than that of a single signal. When the noise intensity reaches a certain value, the noise signal will gradually cover the sine signal, and the value of entropy will gradually approach that of the noise signal. Thus, the IIE curve in which the sine signal is superimposed with white noise tends to rise gradually. With the increase in noise intensity, the IIE value of the sine signal superimposed with sine noise increases first and then decreases. This phenomenon occurs because, when the intensity of sine noise is lower than that of the sine signal, the randomness is enhanced after the superposition. When the intensity of sine noise exceeds that of sine signal, the sine noise dominates, and the value of entropy approaches that of the sine noise gradually; that is, it begins to decline.

### 2.3. Dynamics Mutation Simulation of Time Series Based on IIE

**Figure 3:**
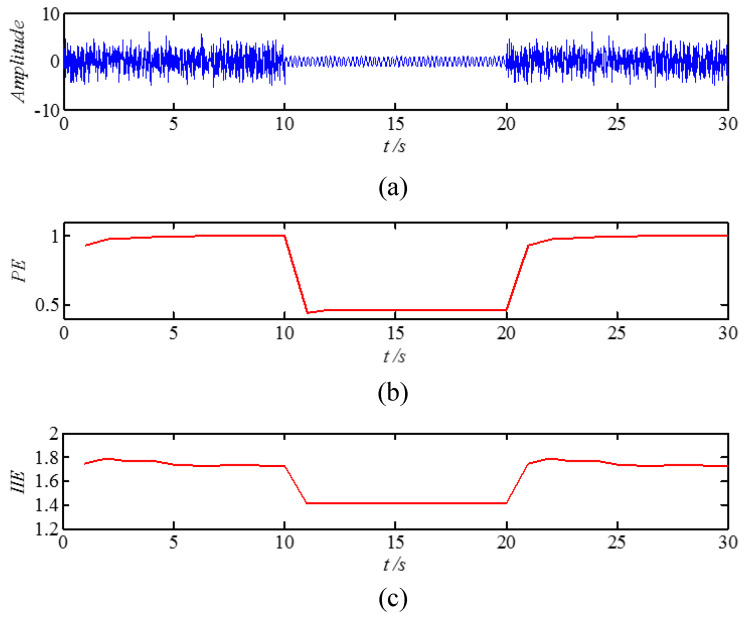
Entropy change chart of the simulation signal. (**a**) original signal; (**b**) permutation entropy (PE); (**c**) improved information entropy (IIE).

In this section, we test whether the IIE algorithm is reasonable for detecting the dynamic mutation in vibration signal analysis. First, a set of simulation signals is presented. The simulation signal is a set of sinusoidal signals, which is superimposed with a white noise signal at the times of 0–10 s and 20–30 s, respectively, and is shown in Figure 3a. Then, the PE and IIE of the signal are calculated. When calculating the PE, the parameter of the embedded dimension is m=5 and the time delay is τ=2 [37]. The computational results of the two entropies are shown in Figure 3b,c. Figure 3c shows that, after the noise signal is superimposed on the sinusoidal signal at the time slice of 0–10 s, the IIE is very large and approaches 1.8, and the volatility is small. This finding indicates that the signal is in a completely random state. When the noise disappears, the signal IIE decreases immediately. At the mutation point of the signal, the IIE jumps. At the mutation point of time 20 s, the noise is superimposed on the original signal again. The signal returns to a disordered state, and the IIE rises immediately to a high value and stabilizes. The PE value exhibits the same variation trend of the signal as the IIE does, as shown in Figure 3b. Thus, if the signal for a given random time series has a sudden change or changes the original state, then the IIE value will show an evident mutation at the corresponding time point. Therefore, the signal simulation analysis demonstrates that the IIE algorithm can detect mutations and perform state recognition. This characteristic of the IIE algorithm allows for its utilization in signal analyses of fault diagnosis.

### 2.4. Running Time of the IIE Compared with that of the PE

The PE and IIE are analyzed in terms of running time. In calculating PE, we need to consider and set three parameters, namely, the length of time series *N*, embedded dimension *m*, and time delay *τ*. Moreover, the determination of *m* and *τ* still depends on experience and on the attempt, which is a bottleneck problem for the PE in engineering applications. However, in calculating IIE, we only need to consider the length of time series *N*. The effect of embedding dimension and time delay on runtime in PE is illustrated in Table 1, where the PE is calculated by selecting dimension *m* = 1–7 under time delay τ=1−4. Table 1 shows that, when less embedded dimension and time delay are used, the running time is short. However, in the practical application of mechanical vibration signals, the validity of calculation results will be affected. Cao et al. emphasized that, when *m* < 4, the signal mutation cannot be accurately detected; when *m* > 7, the reconstructed phase space will homogenize the signal, and abrupt changes in the signal can be detected effectively but with difficulty; when τ > 5, small changes in the signal cannot be detected [37]. If the parameter selection is too small or too large, then the validity of the calculation result will be affected. Moreover, if the parameter selection is too large, then the calculation amount will also be large.

A Gaussian white noise signal with a noise length of 1000 and a noise intensity of 10 dB are used to calculate the running time of PE and IIE separately. Specifically, the PE has embedded dimension *m* = 5 and time delay τ=2. The computation cost of PE is approximately 0.090 s, and the computation cost of IIE is nearly 0.028 s. The algorithms are run using the MATLAB R2014b (MATLAB 8.4, The Mathworks, Inc., Natick, MA, USA) platform on a laptop with a 3.2 GHz AMD Ryzen 5 1400 CPU and 4.0 GB RAM. The calculation results show that the running time of IIE is shorter than that of PE.

## 3. Application Cases Using IIE for Defect Diagnosis of Bearings

### 3.1. Rolling Bearing Fault Diagnosis Based on IIE

If the fault signal is weak during rolling bearing failure, then it is easily covered by other signals (noise). Therefore, extracting feature vectors from fault signals is particularly critical. During the operation of the bearing, the vibration signal easily exhibits non-stationary and nonlinear characteristics. Besides, in the working process of the bearing, the vibration signal may couple with its own nonlinear factors due to the sudden situation and the randomness. In practice, the vibration signal received by the signal acquisition device is usually a one-dimensional time series. When the fault point of the rolling bearing occurs in different parts, the vibration trend of the one-dimensional vector is different. This characteristic can be utilized to trace the health state of the bearing system. Therefore, the IIE algorithm proposed above for rapid detection of vibration signal saltation is considered to be applied in bearing fault diagnosis experiments.

**Figure 4:**
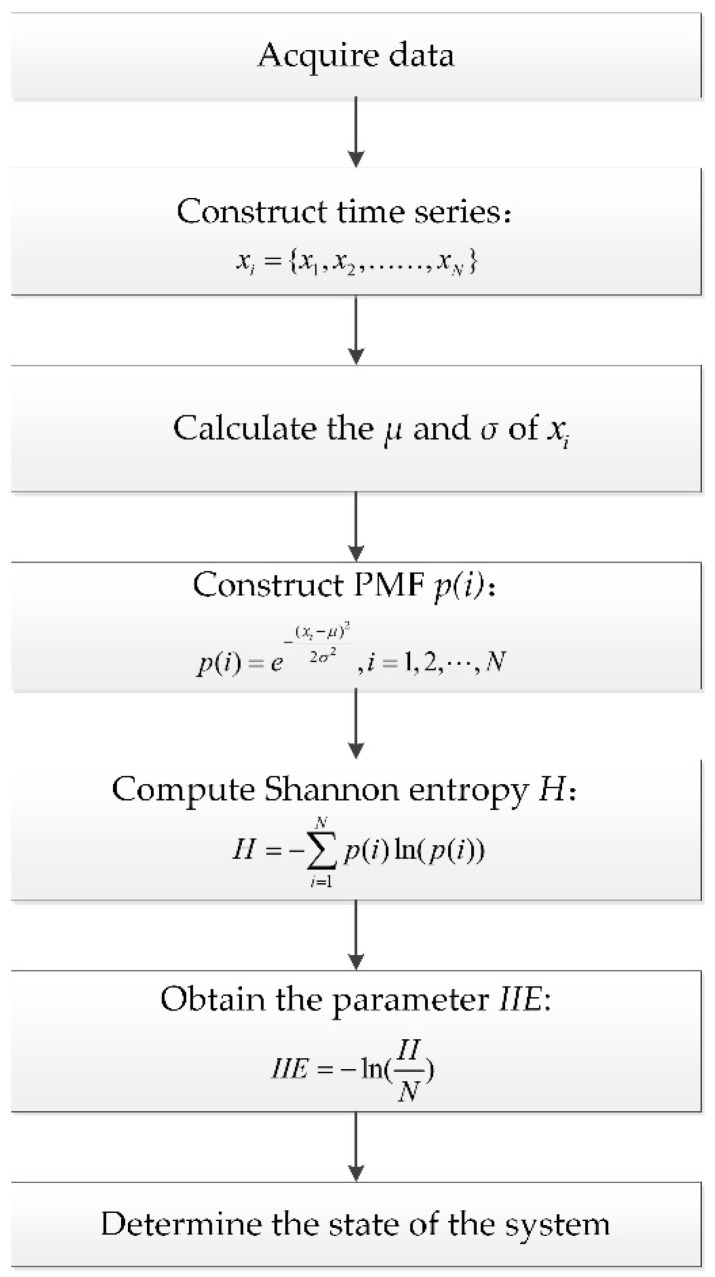
Flowchart of bearing fault diagnosis based on IIE.

The computational flowchart of IIE is shown in Figure 4. Overall, the bearing fault diagnosis based on the IIE algorithm can be summarized as follows:

(1) Acquire data.
(2) Constructing time series: The vibration signals collected from rolling bearings are constructed into time series $x_{i}$.
(3) Calculating the μ and σ of time series $x_{i}$.
(4) Constructing the PMF *p*(*i*).
(5) Computing the Shannon entropy *H*.
(6) Obtaining the parameter of IIE by normalizing *H* with data length *N*.
(7) Determining the state of the system.

In accordance with the results of the IIE analysis, the state of the system operation is judged.

### 3.2. Experimental Result Analysis

**Figure 5:**
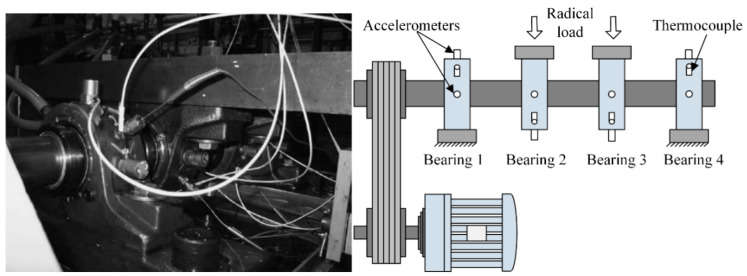
The bearing test platform.

**Figure 6:**
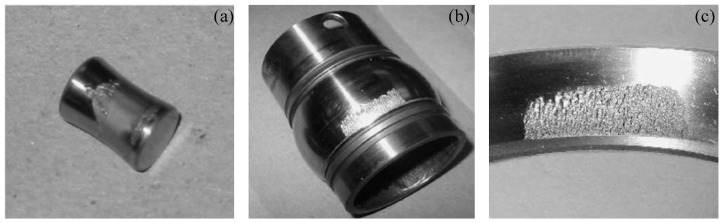
The tested bearing with different fault types: (**a**) Roller element defect, (**b**) inner race defect, and (**c**) outer race defect.

**Case I**: To verify the effectiveness of the proposed method, the experiment is implemented for the bearing vibration signal of the whole lifecycle; all data sets are obtained from the Intelligent Maintenance System Center [38]. The test platform is shown in Figure 5. In the test system, four Rexnord ZA-2115 bearings (Rexnord Corporation, Milwaukee, WI, USA) are mounted on the same test shaft. They are rotated at 2000 rpm by an alternating current motor. Then, a radial load of 6000 pounds is added to the bearing. Four PCB 353B33 accelerometers (PCB Piezotronics, Inc., Buffalo, NY, USA) are installed on the bearing to collect vibration signals. The sampling rate and data length are set to 20 kHz and 20,480 points, respectively. Simultaneously, the NI DAQ card -6062E data acquisition card is used to record the vibration signal every 10 min. Magnetic screw plugs are installed in the oil feedback pipeline. As the bearings wear away, metal debris is gradually absorbed on the magnetic screw plugs. After exceeding a certain level, the machine stops running. At the end of the test, inner race, roller element, and outer race defects occur in bearings 3 and 4 of test 1 and bearing 1 of test 2. Bearing parts with different defects are shown in Figure 6. Additional details of the experiment can be found in Reference [39].

**Figure 7:**
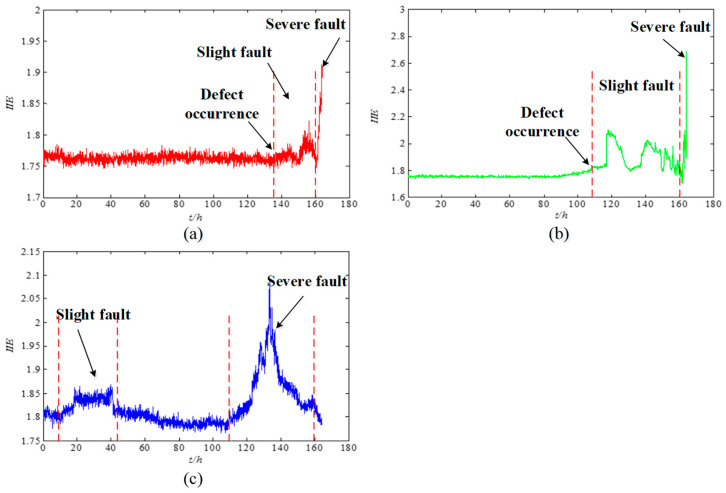
The IIE trend charts of vibration signals from the NASA website: (**a**) Inner race, (**b**) outer race, and (**c**) roller element.

Figure 7 depicts the IIE trend charts of the whole lifecycle signals with different bearing failures. As shown in Figure 7a, the inner race of the bearing is relatively stable before 136 h. When the bearing runs to 136 h, its vibration signal shows a slight jump. This condition indicates that the bearing has begun to appear abnormal. From 136–160 h, the vibration signal fluctuates up and down. Therefore, the bearing is running with a fault, but the fault is not serious. When the bearing runs for more than 160 h, its vibration signal changes drastically, and its entropy value reaches the maximum in 163.3 h. At this time, the bearing has experienced serious failure and reached its life limit. As shown in Figure 7b, the outer race bearing is relatively stable before 108 h. When the bearing runs to 108 h, its vibration signal shows a slight jump. This condition indicates that the bearing has begun to appear abnormal. From 108–160 h, the vibration signal fluctuates up and down. Therefore, the bearing is running with a fault, but the fault is not serious. When the bearing runs for more than 160 h, its vibration signal changes drastically, and its entropy value reaches the maximum in 163.3 h. At this time, the bearing has experienced serious failure and reached its life limit. The whole life analysis diagram of the roller element is shown in Figure 7c. The graph shows that the fluctuation trend between 10–45 h and 110–160 h can be explained by the nature of the damage propagation process. When operating between 10 and 45 h, the roller element surface defect has begun, thereby forming a small flake or crack and then gradually smoothing by continuous rolling contact. From 110–160 h, the roller element surface defect extends to a wide range, and the vibration level rises again. At this time, the bearing has experienced a serious fault to reach its life limit. This phenomenon of “healing” can be confirmed in Reference [39].

**Figure 8:**
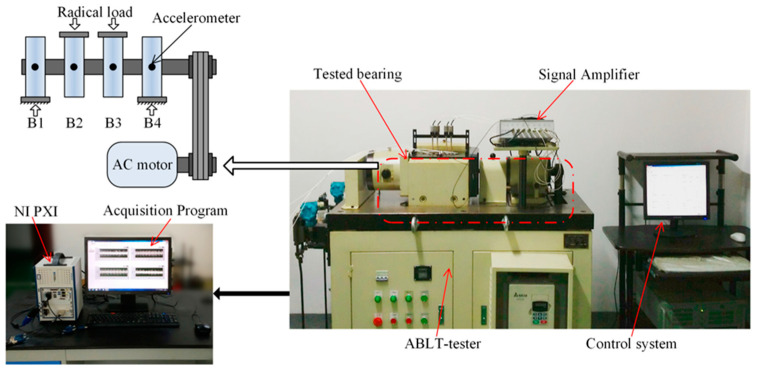
Designed bearing test platform.

**Figure 9:**
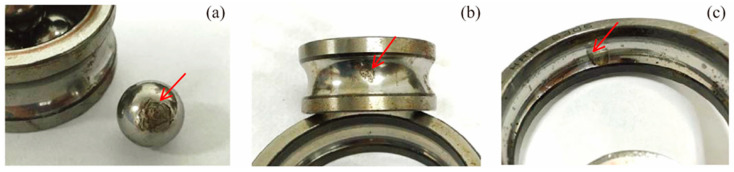
The tested bearing with different fault types: (**a**) Roller element defect, (**b**) inner race defect, and (**c**) outer race defect.

**Case II**: Apart from the IIE extraction and analysis of the faulty bearing data provided by the NASA website, this study also analyzes the bearing life data obtained by our laboratory. The experimental platform, as shown in Figure 8, includes ABLT test platform, signal amplifier, monitoring system, four tested bearings, NI PXI acquisition system, and signal acquisition program based on LabVIEW 2013 (National Instruments Corporation, Austin, TX, USA). The ABLT test platform comes from Hangzhou Bearing Test and Research Center and is composed of but not limited to a transmission system, load system, and lubrication system. The bearings used in the experiment are HRB 6305 (Harbin bearing manufacturing company, Harbin, China). They are mounted on the same experimental shaft and connected to the motor through a belt, which is powered by a three-phase motor. During the operation, the bearings are added with a radial load of 750 kg to hasten the defect occurrence. The rotating speed of the shaft and the sampling frequency are set as 3000 rpm and 20 kHz, respectively. The vibration signals of each bearing are acquired every 5 min using the LABVIEW acquisition program. After a long period of fatigue tests, different types of bearing failures have been obtained, as shown in Figure 9.

**Figure 10:**
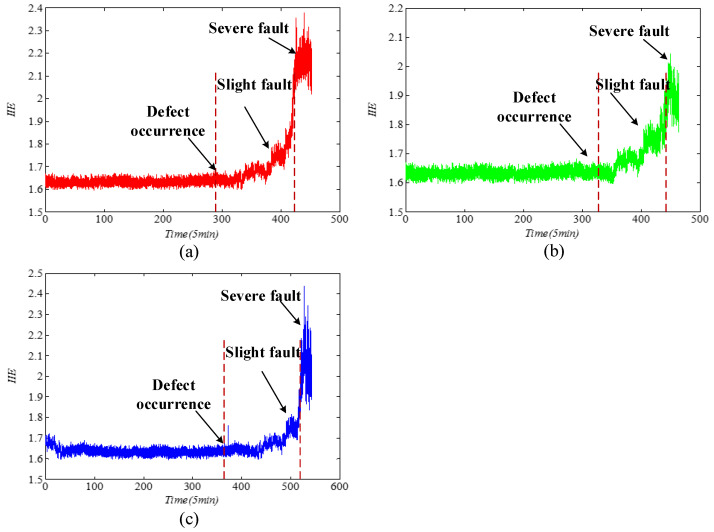
The IIE trend charts of vibration signals from our laboratory: (**a**) Inner race, (**b**) outer race, and (**c**) roller element.

The acquired experimental data are analyzed to verify the proposed IIE feature extraction method for the fault diagnosis of the bearing. The fault vibration signals of the bearing inner ring, bearing outer ring, and rolling element are usually used as analysis objects in validating bearing performance degradation. As shown in Figure 9, the bearing runs continuously for more than 40 h by applying a radial load to accelerate its fatigue failure. Calculation results of the IIE of the whole life vibration signals of bearing in Figure 10a–c show that the bearing performance degradation curves of the inner race, outer race, and roller element extracted from IIE model have good monotonicity and stable change trends. The whole lifecycle of the bearings can be clearly shown from healthy status to minor failures and the serious failures of the degradation process. Therefore, the proposed IIE can be used for performance degradation indicator extraction. This method is sensitive for early fault detection, which is crucial for setting an early alarm for timely maintenance and prevention of cascading failure.

## 4. Conclusions

A sensitive health indicator extraction is crucial for health monitoring and timely maintenance of bearings. This study presents a sensitive feature extraction method based on IIE and applies this method to the health evaluation of different fault bearings. We define the characteristic parameter of IIE by calculating the probability mass function and Shannon entropy of a set of time series. This parameter can effectively distinguish between typical signals and is quite sensitive to signal mutation. Thus, the IIE feature is extracted to conduct the bearing fault diagnosis experiments. The experimental results with different bearing fault cases show that the proposed method can reflect the decline in bearing monotonic performance. The IIE feature extraction can provide information on the entire process of failure occurring and gradually worsening during the operation period of the bearing. The proposed prediction method of bearing performance degradation not only can select few parameters but also can quickly detect the variation in weak signals. Therefore, the method is effective for evaluating the performance degradation of bearings.

## Figures and Tables

**Table 1:** Running time under different embedded dimensions and time delays.

τ\m	1	2	3	4	5	6	7
1	0.008	0.008	0.009	0.023	0.093	0.518	3.615
2	0.003	0.006	0.009	0.022	0.090	0.512	3.515
3	0.003	0.006	0.009	0.022	0.088	0.510	3.545
4	0.003	0.006	0.009	0.022	0.088	0.533	3.432
